# Effects of environmental and genetic interactions on job burnout in coal miners: interactions between occupational stress, coping styles, and *NR3C2* gene polymorphisms

**DOI:** 10.3389/fpubh.2023.1237843

**Published:** 2023-11-20

**Authors:** Xin Lin, Xiaofan Ma, Xiaoting Yi, Chao Qu, Fuye Li

**Affiliations:** Department of Public Health, Xinjiang Medical University, Urumqi, China

**Keywords:** job burnout, occupational stress, coping styles, *NR3C2* gene, GMDR

## Abstract

**Objectives:**

To investigate the current situation regarding occupational burnout among coal miners, explore the relationship between *NR3C2* gene polymorphism and occupational burnout, and analyze the influence of the interaction between environment and gene on occupational burnout. This study provides a scientific basis for formulating health strategies to combat job burnout.

**Methods:**

A total of 1,500 first-line coal mine workers were selected by cluster random sampling, and the job burnout scale, job content questionnaire (JCQ), and simplified coping style questionnaire (SCSQ) were used for the questionnaire survey. A total of 150 workers were randomly selected from the high burnout group and the low burnout group, and a total of 300 workers were selected as the research objects to examine the relationship between gene polymorphism, environment-gene interactions and burnout. This study employed iMLDR^TM^ genotyping technology for *NR3C2* gene (rs5522, rs2070950) polymorphism analysis. The relationship between the occurrence of job burnout, occupational stress, coping styles and the *NR3C2* gene was analyzed.

**Results:**

Finally, a total of 1,282 valid questionnaires were retrieved, with an effective recovery rate of 85.5%. The study included 128 participants (10%) with zero burnout, 400 (31.2%) with mild burnout, 649 (50.6%) with moderate burnout and 105 (8.2%) with severe burnout. There were significant differences in the rate of burnout among miners with respect to sex, age, working years, educational level, shifts, and marital status (*P* < 0.05). The difference in occupational stress between the different job burnout groups was statistically significant (*P* < 0.05). Compared with the GG genotype of rs2070950 of the *NR3C2* gene, the CC genotype was identified as a susceptibility gene for occupational burnout (*P* < 0.05). In respect to rs5522, rs2070950, occupational stress, positive coping, and negative coping, the low-risk group was unlikely to suffer from job burnout compared with the high-risk group (*OR* = 0.103, 95%*CI*: 0.058–0.182).

**Conclusion:**

In addition to demographic characteristics, occupational stress and negative coping styles were also identified as risk factors for job burnout. The interaction between locus rs5522, locus rs2070950, occupational stress, positive response, and negative response were found to affect the incidence of occupational burnout.

## 1 Background

Nowadays, with fierce job competition, tense interpersonal relationships, higher technical requirements and levels of work intensity, the incidence of job burnout has aroused widespread concern in the scientific community (1). Research on burnout began with Freudenberger (2) and Maslach (3), among others. It refers to emotional exhaustion, personality disintegration, and a decreased sense of accomplishment, and can occur in the service industry or medical field in response to excessive working hours, too much work, and working too intensely, ultimately bringing about a series of physiological and psychological changes in the individual (4). Burnout has been found to be a significant predictor of hypercholesterolemia, type II diabetes, coronary heart disease, cardiovascular disease, sexual dysfunction, musculoskeletal disorders, headaches, and gastrointestinal problems, and people with burnout also exhibit a chronic decline that includes profound fatigue, insomnia, dizziness, nausea, allergies, dyspnea, menstrual irregularities, swollen glands, sore throats, recurrent colds, and in some cases, more severe gastrointestinal problems, ulcers, and hypertension (5–12). Job burnout can affect employees' turnover intention through the intermediary role of organizational commitment or job satisfaction. Higher levels of job burnout show a stronger relationship with turnover intention. Moreover, job burnout may also lead to a decline in the quality of employees' work (13–16).

The factors influencing the occurrence of burnout are generally determined by two factors: environmental factors and genetic factors. The environmental influences are work-related factors (17–19), individual characteristics (20–22), and moderating factors (23–25). It has been suggested that burnout may be closely related to the Hypothalamic-Pituitary-Adrenal axis (HPA) (26). The regulation of the HPA axis mainly depends on glucocorticoid receptor (GR) and mineralocorticoid receptor (MR). Li's (27) previous study showed a close relationship between glucocorticoid receptor and job burnout; for example, the rs41423247 locus of the GR gene is a susceptible genotype for burnout compared to the CC genotype (*OR* = 4.00, 95%*CI*: 1.845–8.671); the rs17209237 locus of the GR gene is a susceptible genotype for burnout compared to the GG genotype (*OR* = 2.674, 95%*CI*: 1.346–5.312); and the AA genotype has been identified as a susceptible genotype for burnout (*OR* = 2.674, 95%*CI*: 1.346–5.312). The CT genotype is a susceptible genotype for burnout compared to the CC genotype in the rs258747 locus of the GR gene (*OR* = 1.923, 95%*CI*: 1.1.252–2.954) (28). The gene product of nuclear receptor subfamily 3 group C member 2 (*NR3C2*), salt corticosteroid receptors, is distributed in brain regions mainly in the hippocampus and amygdala, while *NR3C2* has a role in determining the activation threshold of the hypothalamic-pituitary-adrenergic axis (HPA axis). The *NR3C2* is an important gene involved in stress response and is closely related to the anxiety generation and regulation (29, 30). Van Leeuwen et al. (31) investigated the relationship between the rs5522 locus and the stress response, and found that its haplotype may contribute to individual differences in chronic stress perception as well as neuroendocrine and cardiovascular stress responses. The study reported high polymorphic information at the rs2070950 and rs5522 loci, and therefore, served as the genetic polymorphic loci for this study.

China is currently the world's largest producer and consumer of coal (32), with coal resources accounting for 95% of the total domestic fossil energy resources. Coal mine workers work in a special environment, and are subjected to difficult working conditions and psychological pressure. Years spent working in this type of poor environment can easily contribute to the onset of pneumoconiosis, chronic obstructive pulmonary disease, musculoskeletal disorders and other diseases among coal workers (33, 34). In addition, the underground environment of coal mines is very complicated. Water, fire, gas, and coal dust always threaten the lives and safety of coal miners, and cause great psychological pressure. Long-term shift work in coal mines has a sizeable negative impact on the body and mind (35).

The relationship between genes and diseases has become a hot topic in the field of life science. This study explored the relationship between occupational stress, coping styles, *NR3C2* gene polymorphism interaction and job burnout in coal miners, and provided scientific basis for revealing individual differences in job burnout. This study provides a new strategy to screen the population susceptible to job burnout, and provides a scientific basis for coal mining enterprises and the government to propose a reasonable employment system, improve the working conditions of coal miners, and prevent and treat job burnout. Moreover, this study provides a scientific basis for the career choice of individuals who want to work in coal mining related fields.

## 2 Measures

### 2.1 Subjects

This study was approved by the ethics committee of Xinjiang Medical University (No.20160218-109). First, we randomly sampled whole groups to select five coal mining enterprises in Xinjiang province between 2019 and 2020, including one large coal mining enterprise (annual output >1.2 million tons), two medium-sized coal mining enterprises (annual output < 900,000 tons), and two small coal mining enterprises (annual output < 300,000 tons). The target population in this study included a total of 1,500 coal miners workers in Xinjiang who mainly worked in coal mining, excavation, and transportation. Finally, a total of 1,282 valid questionnaires were retrieved, with an effective recovery rate of 85.5%, including 505 questionnaires completed by workers from a large coal mining enterprise, 494 workers from medium-sized coal mining enterprise, and 283 people from a small coal mining enterprise. Before the survey began, all respondents voluntarily provided their written informed consent. The inclusion criteria were: (1) formal employment in a coal mine; (2) aged 18 years or older; length of service ≥1 year; and (3) no blood relationship between the subjects. The exclusion criteria were: (1) a history of genetic disease and mental illness; (2) subjects had a blood disease; and (3) intake of drugs (tricyclic antidepressants, glucocorticoids, dexamethasone, and female hormones) within a week. A total of 300 coal miners, consisting of 150 workers with moderate/severe burnout (high-burnout group) and 150 workers with zero/mild burnout (low-burnout group), were selected for collection of laboratory samples for the study based on a simple random sampling method. Both groups were matched 1:1 with regard to age, gender, ethnicity, type of work, and education level.

### 2.2 Job burnout measurement

We measured job burnout according to the Dai's job burnout questionnaire revised by Li (36, 37). The questionnaire was selected based on the internationally used Maslach Bumout Inventory (MBI), revised by Boles et al. (38) and translated into Chinese by Dai et al. (39), and its reliability and validity were tested. Notably, the scale has satisfactory reliability and validity. The scale includes three dimensions (emotional exhaustion, personality disintegration, and decreased sense of accomplishment) and 15 questions (five questions for each dimension). The questionnaire was scored using a seven-point Likert-type scale (1, “never”; 7, “every day”), while some questions (3, 6, 9, 12, and 15) were reverse scored. Job burnout levels were divided into zero burnout (all below three thresholds), mild burnout (only above one threshold), moderate burnout (only above two thresholds), and severe burnout (all above three thresholds) based on 25 scores for emotional exhaustion, 11 scores for personality disintegration, and 16 scores for personal achievement.

### 2.3 Occupational stress measurement

The Job Content Questionnaire (JCQ) is a concise work-stress questionnaire based on type of job requirements, work autonomy, and social support. This questionnaire was independently developed by Dai (40) and includes three dimensions consisting of five items for psychological demand (PSD), nine items for decision latitude (DL), and eight items for social support (SS). It contains 22 entries in total, and each entry is rated according to a five-point Likert scale. This questionnaire has been shown to have good reliability and validity in other domestic studies (41). The stress level was evaluated by the ratio of the average score between job requirements and work autonomy (Demand/Control Ratio, DCR). A ratio of >1 indicated high occupational tension, and the ratio of < 1 indicated low occupational tension.

### 2.4 Coping style measurement

The Simplified Coping Style Questionnaire (SCSQ) prepared by domestic scholar Xie (42) was adopted. The questionnaire consists of 20 items and can be divided into two dimensions: positive coping styles (12 items reflecting the characteristics of respondents' positive coping styles) and negative coping styles (eight items reflecting the characteristics of respondents' negative coping styles). The questionnaire was graded from 0 to 3. Higher scores for positive coping indicated that respondents were more likely to adopt positive coping styles. Higher scores for negative coping indicated that respondents were more likely to adopt negative coping styles. The Cronbach's alpha coefficient of the positive coping scale was 0.89, whereas the Cronbach's alpha coefficient of the negative coping scale was 0.78.

### 2.5 Genotyping

Based on previous studies and according to the International Haplotype Map Project (https://www.genome.gov/10001688/international-hapmap-project), the Haploview software package (http://www.broad.mit.edu/mpg/haploview) produced a MAF (minimum allele frequency) greater than 0.1, while the linkage disequilibrium parameter *r*^2^ was greater than 0.8. We then used the SNaPshot method to screen candidate genes and tag SNPs associated with emotion (*NR3C2* rs5522 and rs2070950 SNPs). PCR Primers (Table 1) were found from related literature. The selected rs5522, rs2070950 were genotyped using the multiple high-temperature ligase detection reaction technology (iMLDR^TM^) by Denesky Biotechnologies, Inc. (Shanghai, China) (43).

**Table 1:** The list of primers for PCR amplification.

**Gene**	**Gene site**	**Direction**	**Primer**
*NR3C2*	rs5522	F(5′-3′)	CTGCAAACAGACGGGCTTTTCT
		R(3′-5′)	TGAACACGCCCTTGAGATCATT
	rs2070950	F(5′-3′)	GGAGACTGTGGTAGCCTTTGGTCTC
		R(3′-5′)	TGGAAGGCAGCTTCTATATGATCCA

### 2.6 Reagents and instruments

Cell-cracking solution CL, protease K, Buffer FG, eluting buffer TB (TIANGEN), Microsampler Eppendorf, desktop high-speed centrifuge TD5A-WS, Ultra-micro photometer NanoDeop 2000, etc. PCR amplification: PCR MyCycler, Gel imager MyCycler, Nucleic acid dyes (Biomed Company), PCR reaction buffers, MgCl2 (Takara Company), QT-1 vortex mixer(Shanghai Kit Analytical Instrument Co., LTD), Mini-4lc microcentrifuge (Zhuhai Black horse Medical Instrument Co., LTD), TD5A-WS desktop low speed centrifuge (Changsha Xiangyi centrifuge Instrument Co., LTD), Gel imager (Shanghai Peiqing Technology Co., LTD), FR-110 UV analysis device(Shanghai Furi Technology Co., LTD), FR-250 electrophoresis apparatus (Shanghai Furi Technology Co., LTD), 3730xl genetic analyze (ABI).

### 2.7 Quality control

The investigators involved in this study underwent uniform training, while the coal miners were instructed to complete the Occupational Health Survey scale. Blood samples drawn by a nurse from a hospital specialized in occupational diseases were collected promptly to prevent repeated dissolution. DNA extraction was performed according to the steps required by blood Genomic DNA Extraction Kit. The genotypes of the selected loci were determined in strict accordance with the operation procedure of iMLDR^TM^. When testing in the laboratory, a familiar experimental instrument with the specification was used to ensure that the instrument was in good working condition. Samples and reagents were kept by strictly conforming to the quality and normal standards of molecular biology laboratories to ensure that they were not contaminated. A random sample of 5% of the high-burnout group and the low-burnout group was genotyped by two different researchers with a 99% repetition rate.

### 2.8 Statistical analysis

All data were entered into the Epidata 3.1 database and checked for data consistency. The data were analyzed using IBM SPSS statistical software (version 21.0). Quantitative data are expressed as mean ± standard deviation, and the mean of the two groups were compared by performing two independent samples *t*-tests. In addition, allele and genotype distribution frequency of the high-burnout group and the low-burnout group were analyzed by single-factor analysis, as well as Odds Ratios (OR) and 95% Confidence Intervals (95%CI). Regression models of rs5522 and rs2070950 of the *NR3C2* gene were established using R software. In the regression model, covariates with more than 10% influence on the regression coefficient of each gene and covariates with *P* < 0.1 regression coefficient of each SNP are confounding factors. Consequently, sex and working years were identified as confounding factors. After adjusting for confounding factors, SPSS 21.0 software was used to analyze the distribution of SNP genotypes and alleles in the job burnout case group and control group, and the correlation between SNP genotypes and the occurrence of job burnout was analyzed by *OR*s and 95%*CI*s. Gene-environment interaction analyses were performed using Generalized Multi-factor Dimensionality Reduction (GMDR) software. A *P*-value of 0.05 was considered statistically significant.

## 3 Results

### 3.1 Job burnout among participants with different demographic characteristics

This study included 128 participants (10%) with zero burnout, 400 (31.2%) with mild burnout, 649 (50.6%) with moderate burnout and 105 (8.2%) with severe burnout. Differences in sex, age, length of service, educational level, shift system, and marital status were statistically significant (*P* < 0.05; Table 2).

**Table 2:** Occupational burnout degree of coal miners with different individual characteristics, *n* (%).

**Variables**	**Sort**	**Burnout level**				**χ^2^**	***P***
		**Zero burnout**	**Mild burnout**	**Moderate burnout**	**Severe burnout**		
**Sex**							< 0.001
	Male	109 (9.1)	370 (30.8)	624 (52.0)	98 (8.2)	23.352	
	Female	19 (23.5)	30 (37.0)	25 (30.9)	7 (8.6)		
**Age group, years**							< 0.001
	≤ 35	55 (11.7)	162 (34.3)	228 (48.3)	27 (5.7)	29.483	
	35~	41 (10.3)	131 (32.8)	204 (51.1)	23 (5.8)		
	>45	32 (7.8)	107 (26.0)	217 (52.8)	55 (13.4)		
**Working years**							0.011
	≤ 10	84 (10.4)	271 (33.6)	397 (49.3)	54 (6.7)	11.188	
	>10	44 (9.2)	129 (27.1)	252 (52.9)	51 (10.7)		
**Educational level**							0.040
	Junior high school or below	56 (7.8)	224 (31.3)	383 (53.5)	53 (7.4)	13.175	
	High school	24 (11.1)	65 (30.1)	108 (50.0)	19 (8.8)		
	College, secondary school and above	48 (13.7)	111 (31.7)	158 (45.1)	33 (9.4)		
**Type of work**							0.837
	Coal miner	15 (8.5)	58 (32.8)	89 (50.3)	15 (8.5)	2.767	
	Excavation worker	22 (10.7)	56 (27.2)	108 (52.4)	20 (9.7)		
	Other	91 (10.1)	286 (31.8)	452 (50.3)	70 (7.8)		
**Shift**							0.003
	Fixed day shift	45 (13.6)	116 (35.2)	151 (45.8)	18 (5.5)	14.298	
	Shift	83 (8.7)	284 (29.8)	498 (52.3)	87 (9.1)		
**Marital status**							0.003
	Single	19 (12.8)	63 (42.3)	59 (39.6)	8 (5.4)	19.876	
	Married	108 (9.6)	335 (29.8)	586 (52.2)	94 (8.4)		
	Divorced and Widowed	1 (10.0)	2 (20.0)	4 (40.0)	3 (30.0)		
**Monthly income**							0.712
	≤ 7	58 (11.3)	156 (30.3)	261 (50.7)	40 (7.8)	3.738	
	7~	53 (8.7)	190 (31.1)	316 (51.8)	51 (8.4)		
	>10	17 (10.8)	54 (34.4)	72 (45.9)	14 (8.9)		

### 3.2 Comparison of job burnout scores for different demographic characteristics

The results showed differences in overall job burnout scores according to the participants' sex, age, length of service, educational level, type of work, shift system, and marital status (*P* < 0.05; Table 3).

**Table 3:** Scores of different dimensions of occupational burnout of coal miners with different individual characteristics ($\bar{x}$ ± s).

**Variables**	**Sort**	**Emotional exhaustion**	**Personality disintegration**	**Decreased sense of accomplishment**	**Job burnout**
**Sex**					
	Male	17.25 ± 7.31	15.21 ± 7.23	20.17 ± 7.74	52.64 ± 14.59
	Female	14.67 ± 6.70	12.10 ± 6.15	18.37 ± 7.53	45.14 ± 14.80
	*T*	3.101	4.362	2.032	4.477
	*P*	0.002	< 0.001	0.042	< 0.001
**Age group, years**					
	≤ 35	16.73 ± 6.96	13.67 ± 6.46	19.39 ± 6.97	49.78 ± 12.89
	35~	16.41 ± 6.99	14.68 ± 7.05^a^	20.12 ± 7.88	51.21 ± 14.38
	>45	18.17 ± 7.84^ab^	16.89 ± 7.76^ab^	20.78 ± 8.36^a^	55.84 ± 16.25 ^ab^
	*F*	6.869	23.477	3.588	20.450
	*P*	0.001	< 0.001	0.028	< 0.001
**Working years**					
	≤ 10	16.76 ± 7.12	14.61 ± 7.09	19.88 ± 7.57	51.25 ± 14.29
	>10	17.65 ± 7.56	15.71 ± 7.35	20.36 ± 8.02	53.72 ± 15.29
	*t*	2.123	2.637	1.059	2.905
	*P*	0.034	0.008	0.290	0.004
**Educational level**					
	Junior high school or below	16.75 ± 7.45	15.59 ± 7.43^c^	20.53 ± 8.06^c^	52.87 ± 14.98
	High school	16.88 ± 7.04	15.25 ± 7.54 ^c^	20.52 ± 7.90^c^	52.65 ± 15.42
	College, secondary school and above	17.91 ± 7.09	13.71 ± 6.31	18.81 ± 6.78	50.43 ± 13.56
	*F*	3.102	8.263	6.368	3.406
	*P*	0.045	< 0.001	0.002	0.033
**Type of work**					
	Coal miner	17.71 ± 7.23	15.51 ± 6.75	19.14 ± 6.89	52.35 ± 13.77
	Excavator worker	17.11 ± 7.77	16.70 ± 8.30	20.82 ± 8.92	54.62 ± 18.04
	Other	16.97 ± 7.20	14.54 ± 6.96^d^	20.07 ± 7.60	51.57 ± 13.98^d^
	*F*	0.760	8.132	2.249	3.636
	*P*	0.468	< 0.001	0.106	0.027
**Shift**					
	Fixed day shift	16.98 ± 7.18	13.68 ± 6.63	18.78 ± 7.08	49.45 ± 13.03
	Shift	17.13 ± 7.34	15.48 ± 7.34	20.50 ± 7.91	53.11 ± 15.15
	*t*	0.316	3.921	3.486	3.913
	*P*	0.752	< 0.001	0.001	< 0.001
**Marital status**					
	Single	16.20 ± 6.79	13.00 ± 6.65	19.00 ± 6.97	48.20 ± 13.29
	Married	17.17 ± 7.35	15.25 ± 7.21	20.18 ± 7.84	52.61 ± 14.76
	Divorced and Widowed	21.30 ± 7.07	18.50 ± 9.07^e^	21.90 ± 6.56	61.70 ± 19.09^ef^
	*F*	2.850	7.699	1.825	8.107
	*P*	0.058	< 0.001	0.162	< 0.001
**Monthly income**					
	≤ 7	17.06 ± 7.19	14.79 ± 7.24	20.32 ± 7.58^g^	52.18 ± 14.16
	7~	17.02 ± 7.31	15.16 ± 7.10	20.29 ± 7.93^g^	52.47 ± 14.90
	>10	17.46 ± 7.61	15.21 ± 7.53	18.31 ± 7.33	50.97 ± 15.77
	*F*	0.231	0.430	4.621	0.642
	*P*	0.794	0.651	0.010	0.526

^a^Compared with the group aged ≤ 35 years, *P* < 0.05. ^b^Compared with the 35~ group. ^c^Compared with the secondary college and above group, *P* < 0.05. ^d^Compared with the excavator workers, *P* < 0.05. ^e^Compared with the unmarried group, *P* < 0.05. ^f^compared with the married group, *P* < 0.05. ^g^Compared with the >100,000 group, *P* < 0.05.

### 3.3 Analysis of the correlation between job burnout and occupational stress and coping styles

Among the 1,282 respondents surveyed, 822 (64.1%) coal miners had a DCR ≤ 1, while 460 (35.9%) coal miners had a DCR > 1. Job requirements were positively associated with emotional exhaustion and job burnout (*P* < 0.05). Decision autonomy was negatively associated with reduced sense of achievement (*P* < 0.05). Social support was negatively associated with personality disintegration and reduced sense of achievement and job burnout (*P* < 0.05). DCR was positively associated with emotional exhaustion and job burnout (*P* < 0.05). Positive coping was negatively associated with personality disintegration, reduced sense of achievement and job burnout (*P* < 0.05). Negative coping was positively associated with emotional exhaustion, personality disintegration, reduced sense of achievement and job burnout (*P* < 0.05; Table 4).

**Table 4:** Partial correlation analysis between occupational burnout and job content and coping styles (*r*_*s*_).

**Variables**	**Emotional exhaustion**	**Personality disintegration**	**Reduced sense of achievement**	**Job burnout**
Job requirements	0.145^*^	0.034	−0.021	0.078^*^
Decision autonomy	−0.029	−0.004	−0.102^*^	−0.038
Social support	−0.051	−0.060^*^	−0.147^*^	−0.134^*^
DCR	0.134^*^	0.040	0.049	0.113^*^
Positive coping	−0.022	−0.106^*^	−0.291^*^	−0.219^*^
Negative coping	0.184^*^	0.122^*^	0.059^*^	0.121^*^

^*^P < 0.05.

### 3.4 Comparison of occupational burnout in different occupational stress groups

DCR ≤ 1 accounted for 74.2% of the zero burnout group, 66.3% of the mild burnout group, 62.2% of the moderate burnout group, and 55.2% of the severe burnout group. The difference between different burnout level subgroups was statistically significant when compared between DCR subgroups (*P* < 0.05; Table 5).

**Table 5:** DCR group comparison of different occupational burnout degree groups (*n*, %).

**Variables**	***N***	**DCR ≤ 1**	**DCR > 1**	**χ^2^**	***P***
Zero burnout	128	95 (74.2)	33 (25.8)	11.050	0.011
Mild burnout	400	265 (66.3)	135 (33.8)		
Moderate burnout	649	404 (62.2)	245 (37.8)		
Severe burnout	105	58 (55.2)	47 (44.8)		

### 3.5 Comparison of coping styles among different burnout groups

There were significant differences in the scores of positive coping styles and negative coping in different levels of burnout groups (*P* < 0.05; Table 6).

**Table 6:** Comparison of coping styles among different burnout groups ($\bar{x}$ ± s).

**Variables**	**Positive coping**	**Negative coping**
Zero burnout	21.90 ± 6.67	6.91 ± 4.60
Mild burnout	18.88 ± 7.29^a^	7.35 ± 4.69
Moderate burnout	17.59 ± 6.89^a^	8.47 ± 5.00^ab^
Severe burnout	17.13 ± 8.76^ab^	8.59 ± 5.83^ab^
*F*	14.618	6.836
*P*	< 0.001	< 0.001

^a^*P* < 0.05 compared with the zero burnout group. ^b^*P* < 0.05 compared with the mild burnout group.

### 3.6 Hardy-Weinberg equilibrium test

The actual values of rs5522 and rs2070950 loci in this selection of control and case groups matched well with the expected values, and none of the differences were statistically significant (*P* > 0.05), in accordance with the Hardy-Weinberg law of genetic equilibrium (Table 7).

**Table 7:** Hardy-Weinberg genetic balance test.

**Gene**	**Gene locus**	**Genotype**	**Control group**		**χ^2^**	***P***	**Case group**		**χ^2^**	***P***
			**Actual value**	**Expected value**			**Actual value**	**Expected value**		
*NR3C2*	rs5522	CC	6	4.68	0.548	0.760	2	1.93	0.004	0.998
		CT	41	43.64			30	30.15		
		TT	103	101.68			118	117.93		
	rs2070950	GG	18	14.42	1.873	0.392	8	5.80	1.291	0.524
		GC	57	64.17			43	47.40		
		CC	75	71.41			99	96.80		

### 3.7 Analysis of the *NR3C2* gene and the degree of effect of burnout

The distribution of each different genotype and allele of *NR3C2* gene rs2070950 locus was different between the control and case groups and the difference was statistically significant (*P* < 0.05). The difference in the distribution of different genotypes at the rs5522 locus of the *NR3C2* gene between the control and case groups was not statistically significant (*P* > 0.05). Furthermore, the distribution of different allele frequencies at the rs5522 locus was different between control and case groups, and the difference was statistically significant (*P* < 0.05; Table 8).

**Table 8:** rs5522 and rs2070950 genotype and allele distribution analysis of the influence degree of occupational burnout (*n*, %).

**Genetic locus**	**Genetype/allele**	***N***	**Control group**	**Case group**	**χ^2^**	***P***	**OR (95%CI)**
rs5522	CC	8	6 (0.04)	2 (0.01)	4.722	0.094	1.000
	CT	71	41 (0.27)	30 (0.20)			2.133 (0.398,11.434)
	TT	221	103 (0.69)	118 (0.79)			3.306 (0.646,16.915)
	C	87	53 (0.18)	34 (0.11)	4.853	0.028	1.000
	T	513	247 (0.82)	266 (0.89)			1.688 (1.057,2.694)
rs2070950	GG	26	18 (0.12)	8 (0.05)	9.116	0.010	1.000
	GC	100	57 (0.38)	43 (0.29)			1.614 (0.637,4.087)
	CC	174	75 (0.50)	99 (0.66)			2.792 (1.144,6.812)
	G	152	93 (0.31)	59 (0.20)	10.186	0.001	1.000
	C	448	207 (0.69)	241 (0.80)			1.837 (1.258,2.683)

### 3.8 Analysis of gene-gene and environment-gene interactions to burnout

The GMDR model was used to analyze the gene-environment interaction for a total of five factors: rs5522, rs2070950, occupational strain, positive coping, and negative coping. The optimal combination was finally determined, as shown in Table 9, with negative coping as the main effect and an interaction model consisting of rs5522 locus, rs2070950 locus, occupational tension, positive coping and negative coping, which had the greatest cross-validation agreement (10/10), while the highest test sample accuracy was 0.6722 with a *p*-value of 0.0010.

**Table 9:** Occupational burnout GMDR model of environment-gene interaction.

**Model**	**Training sample accuracy**	**Accuracy of test samples**	**Cross-validation consistency**	***P***
X5	0.6367	0.6367	10/10	0.0010
X4‵X5	0.6774	0.6700	10/10	0.0010
X1‵X4‵X5	0.6959	0.6431	6/10	0.0107
X1‵X3‵X4‵X5	0.7304	0.6666	5/10	0.0010
X1‵X2‵X3‵X4‵X5	0.7444	0.6736	10/10	0.0010

X1: rs5522; X2: rs2070950; X3: occupational strain; X4: positive coping; X5: negative coping.

### 3.9 Regression analysis of the effects of gene-environment interactions on job burnout

According to the results of GMDR software analysis, the subjects were divided into low risk group and high risk group. The rs5522 and rs2070950 loci of NR3C2 gene were grouped according to the dominant genetic model and the interaction with environmental factors was analyzed by Logistic regression. The risk of job burnout in CC/CT^*^GG/GC^*^occupational stress negative individuals was lower than TT^*^CC^*^occupational stress positive individuals (*OR* = 0.254, 95%*CI*: 0.117~0.555). Compared with TT^*^CC^*^low positive coping, CC/CT^*^GG/GC^*^moderate positive coping was a protective factor for job burnout (*OR* = 0.245, 95%*CI*: 0.089~0.670). The risk of job burnout in CC/CT^*^GG/GC^*^high positive coping individuals was lower than TT^*^CC^*^low positive coping individuals (*OR* = 0.139, 95%*CI*: 0.034~0.564). Compared with TT^*^CC^*^high negative coping, CC/CT^*^GG/GC^*^low negative coping was a protective factor for job burnout (*OR* = 0.167, 95%*CI*: 0.029~0.956). Compared with the high-risk group in the aspects of rs5522, rs2070950, occupational stress, positive coping, and negative coping, the low-risk group is unlikely to suffer from job burnout (*OR* = 0.103, 95%*CI*: 0.058–0.182; Table 10).

**Table 10:** Environment-gene interaction on occupational burnout of logistic regression analysis.

**Interaction items**	**Compare groups**	**Reference group**	**β**	***Wald***	***OR***	**95%*CI***
rs5522‵rs2070950‵ Occupational stress	CC/CT^*^GG/GC^*^ Occupational stress negative	TT^*^CC^*^ Occupational stress positive	−1.369	11.856	0.254^a^	0.117~0.555
rs5522‵rs2070950‵ Positive coping	CC/CT^*^GG/GC^*^ Moderate positive coping	TT^*^CC^*^ Low positive coping	−1.407	7.501	0.245^a^	0.089~0.670
	CC/CT^*^GG/GC^*^ High positive coping		−1.977	7.609	0.139^a^	0.034~0.564
rs5522‵rs2070950‵ Negative coping	CC/CT^*^GG/GC^*^ Moderate Negative coping	TT^*^CC^*^ High Negative coping	−0.542	0.344	0.582	0.095~3.557
	CC/CT^*^GG/GC^*^ Low Negative coping		−1.792	4.041	0.167^a^	0.029~0.956
rs5522‵rs2070950‵ Occupational stress‵positive coping‵negative coping	Low risk group	High risk group	−2.277	60.676	0.103^a^	0.058~0.182

^a^*P* < 0.05;/, OR; ^*^, AND.

## 4 Discussion

The survey results showed that among the 1,282 respondents, 128 (10.0%) had zero burnout, 400 had mild burnout (31.2%), 649 had moderate burnout (50.6%), and 105 had severe burnout (8.2%). The detection rate of worker burnout was 90.0%. Male coal miners had higher burnout scores than females, consistent with the findings of Gao et al. (44). Compared to female coal miners who work on the surface, male coal miners had a higher risk factor and higher levels psychological stress than females working in the underground working environment. The burnout scores of coal miners in the >45-year-old group were higher than those in the ≤ 35-year-old group and the 35–45-year-old group, consistent with the survey results of Sun et al. (45) on copper-nickel mine workers. The burnout scores of coal miners with a college education, technical secondary school education and above were lower than those of the other two groups, consistent with the survey results of bus drivers reported by Wang et al. (46). With regards to medical staff, divorced, and widowed coal miners had higher burnout scores than unmarried and married workers, which was in line with the findings of Zhang et al. (47). There was no significant difference in burnout scores between married and unmarried groups, consistent with Du and Zhu (48), which may be because divorced and widowed employees have higher stress levels than the other two groups (49), and subjective stress, psychological stress, and physical stress may all increase the level of job burnout (50). Coal miners with economic income >100,000 yuan/year scored lower than the other two groups on the dimension measuring a reduced sense of achievement, which was in agreement with the research results of Liu et al. (51). Workers with a low working ability and high income may have a more positive attitude toward their own working ability. Coal miners with more than 10 years of work experience had higher scores on the dimension corresponding to emotional exhaustion, personality disintegration and occupational burnout than those with less than 10 years of work experience, which was consistent with the research results of Yang et al. (52). The high physical intensity of repetitive labor tends to cause workers to feel disinterested and mentally exhausted. Coal miners in the excavation group scored higher on the personality disintegration dimension and had higher levels of job burnout than the other groups. Participants who worked fixed day shifts had lower burnout scores than coal miners who worked shift work, which was in line with the results of Zhang et al.'s (53) study involving medical staff, and shift work was a risk factor for burnout. This may be because shift work tends to disrupt the normal daily lives of workers. Irregular work and rest affect the physical and mental health of workers (54).

In this survey on the work content of coal miners, coal miners with a DCR > 1 accounted for 35.9%, which was in line with the survey results of coal miners reported by Han et al. (55). The results of the partial correlation analysis showed that job requirements, occupational stress and occupational burnout were positively correlated, while social support was negatively correlated with occupational burnout. Social support is a moderator of occupational stress, and more social support will reduce the level of job burnout (56–58). The results related to coping styles and job burnout showed that positive coping was negatively related to job burnout, while negative coping was positively related to job burnout, which was consistent with the research results of Sun et al. (59). Bogdan et al. (60) found that the rs5522 locus polymorphism affects functional genetic variation in HPA axis function, and functional genetic variation in HPA axis responses to stress may mediate the risk of mental illness. This study selected the rs5522 and rs2070950 loci of the *NR3C2* gene. The results showed that the T allele of the rs5522 locus increased the risk of job burnout (*OR* = 1.688, 95%*CI*: 1.057–2.694). The rs2070950 locus of the *NR3C2* gene was associated with the occurrence of job burnout, and the CC genotype (*OR* = 2.792, 95%*CI*: 1.144–6.812) and C allele (*OR* = 1.837, 95%*CI*: 1.258–2.683) increased the risk of burnout among coal miners.

The results of the gene-environment analysis showed that negative coping was the main effect and the best joint effect model consisting of rs5522, rs2070950, DCR, positive coping, and negative coping factors had the greatest degree of effect on burnout, with a cross-validation consistency of (10/10) and a high test sample precision of 0.6722.

The results showed that the *NR3C2* gene interacted with occupational strain and coping style, and the more significant interaction combinations at high risk of burnout were moderate negative coping, moderate positive coping, positive for occupational strain, TT genotype at the rs5522 locus of the *NR3C2* gene, and CC genotype at the rs2070950 locus of the *NR3C2* gene. This was consistent with the results of Wang et al. (61) who found that the interaction term of coping style and job stress had a regression effect on burnout. Further logistic regression analysis revealed that the environment-gene GMDR interaction results were protective factors for burnout in the low risk group compared to the high risk group (*OR* = 0.103, 95%*CI*: 0.058–0.182), and the rs5522 and rs2070950 loci of the *NR3C2* gene were not interaction main effects, two loci SNPs variants in the interaction increased the risk of burnout. The risk of job burnout in CC/CT^*^GG/GC^*^occupational stress negative individuals was significantly reduced (*OR* = 0.254, 95%*CI*: 0.117–0.555) than in TT^*^CC^*^occupational stress positive individuals. Suggesting that the interaction of occupational stress with rs5522 and rs2070950 locus influenced the level of burnout occurrence. Compared with TT^*^CC^*^low positive coping, CC/CT^*^GG/GC^*^moderate positive coping was a protective factor for job burnout (*OR* = 0.245, 95%*CI*: 0.089~0.670). The risk of job burnout in CC/CT^*^GG/GC^*^high positive coping individuals was lower than TT^*^CC^*^low positive coping individuals (*OR* = 0.139, 95%*CI*: 0.034~0.564). Suggesting that both negative coping and positive coping can interact with the rs5522 and rs2070950 loci of the *NR3C2* gene to influence the level of burnout.

## 5 Conclusion

Mild, moderate, and severe job burnout were detected among 90% of the coal miners in this study. The incidence of job burnout among coal miners was high and mainly concentrated around mild and moderate burnout. Gender, age, shift work, marital status, positive coping, negative coping, job requirements, and social support influenced the level of burnout.

The prevalence of occupational strain among coal miners with the job requirement-autonomy model was average, and higher levels of occupational strain were associated with a higher probability of burnout; positive coping reduced the risk of burnout, whereas negative coping increased the risk of burnout.

The CC genotype at the rs2070950 locus of the *NR3C2* gene was identified as a susceptible genotype for burnout. As for Gene-Environment interaction, the interaction among rs5522 and rs2070950 sites of *NR3C2* gene, occupational stress, positive coping styles and negative coping styles could affect the level of job burnout. The occurrence of job burnout is a complex process. On the one hand, the body's exposure to environmental risk factors may increase the risk of job burnout, on the other hand, the existence of gene polymorphism may make individuals carrying susceptible genes less exposed to environmental risk factors can develop burnout. Therefore, the occurrence of job burnout may be the result of the interaction between environmental factors and genes. Coal mine enterprises and higher management units should strive to ensure a safe working environment, and increase the level of coal mine automation, while health management departments should aim to provide better health care. Management personnel should reasonably allocate the working hours and tasks of the workers and provide them with appropriate supports. Coal mine enterprises should formulate a reasonable system, improve the working environment and conditions, and carry out stress management training programs, such as strengthening communication within the organization and offering psychological counseling, so as to effectively reduce the possibility of job burnout by addressing various factors. Workers themselves should also adopt a positive attitude to reduce the possibility of job burnout.

Our study has several important limitations that need to be considered. Firstly, genetic testing was not conducted on all the samples in this study. Therefore, the results obtained from this study need to be validated in a larger sample study. Secondly, there is a limited number of genotypes included in this study, and there are other genes associated with the HPA axis. To better investigate the relationship between job burnout and genetic and environmental factors, a wider range of genotypes will be included in future studies. Lastly, this study utilized a cross-sectional investigation and research method, which allows for only limited information on the occurrence of the disease. To further explore the impact of gene-environment interactions on burnout, establish a network model of genetic environmental susceptibility factors, and delve into the pathogenesis of burnout, cohort studies will be considered in the future.

## Data availability statement

The original contributions presented in the study are included in the article/supplementary material, further inquiries can be directed to the corresponding author.

## Ethics statement

This study was approved by the Ethics Committee of Xinjiang Medical University (No.20160218-109). The studies were conducted in accordance with the local legislation and institutional requirements. The participants provided their written informed consent to participate in this study.

## Author contributions

XL, XM, and FL conceived and designed the study. XL, XM, XY, CQ, and FL contributed to the acquisition, analysis, and interpretation of data, were involved in drafting the manuscript, and revising it for important intellectual content. All authors contributed substantially to the work presented in this paper. All authors reviewed and approved the final manuscript.
